# Isolation of a high‐affinity Bet v 1‐specific IgG‐derived ScFv from a subject vaccinated with hypoallergenic Bet v 1 fragments

**DOI:** 10.1111/all.13394

**Published:** 2018-02-20

**Authors:** E. Gadermaier, K. Marth, C. Lupinek, R. Campana, G. Hofer, K. Blatt, D. Smiljkovic, U. Roder, M. Focke‐Tejkl, S. Vrtala, W. Keller, P. Valent, R. Valenta, S. Flicker

**Affiliations:** ^1^ Division of Immunopathology Institute of Pathophysiology and Allergy Research Center for Pathophysiology, Infectiology and Immunology Vienna General Hospital Medical University of Vienna Vienna Austria; ^2^ Institute of Molecular Biosciences BioTechMed, University of Graz Graz Austria; ^3^ Division of Hematology and Hemostaseology Department of Internal Medicine I Vienna General Hospital Medical University of Vienna Vienna Austria; ^4^ GE Healthcare Europe GmbH Freiburg Germany; ^5^ NRC Institute of Immunology FMBA of Russia Moscow Russia

**Keywords:** allergy, Bet v 1, combinatorial cloning, IgG antibody, recombinant hypoallergenic allergen derivative

## Abstract

**Background:**

Recombinant hypoallergenic allergen derivatives have been used in clinical immunotherapy studies, and clinical efficacy seems to be related to the induction of blocking IgG antibodies recognizing the wild‐type allergens. However, so far no treatment‐induced IgG antibodies have been characterized.

**Objective:**

To clone, express, and characterize IgG antibodies induced by vaccination with two hypoallergenic recombinant fragments of the major birch pollen allergen, Bet v 1 in a nonallergic subject.

**Methods:**

A phage‐displayed combinatorial single‐chain fragment (ScFv) library was constructed from blood of the immunized subject and screened for Bet v 1‐reactive antibody fragments. ScFvs were tested for specificity and cross‐reactivity to native Bet v 1 and related pollen and food allergens, and epitope mapping was performed. Germline ancestor genes of the antibody were analyzed with the ImMunoGeneTics (IMGT) database. The affinity to Bet v 1 and cross‐reactive allergens was determined by surface plasmon resonance measurements. The ability to inhibit patients’ IgE binding to ELISA plate‐bound allergens and allergen‐induced basophil activation was assessed.

**Results:**

A combinatorial ScFv library was obtained from the vaccinated donor after three injections with the Bet v 1 fragments. Despite being almost in germline configuration, ScFv (clone H3‐1) reacted with high affinity to native Bet v 1 and homologous allergens, inhibited allergic patients’ polyclonal IgE binding to Bet v 1, and partially suppressed allergen‐induced basophil activation.

**Conclusion:**

Immunization with unfolded hypoallergenic allergen derivatives induces high‐affinity antibodies even in nonallergic subjects which recognize the folded wild‐type allergens and inhibit polyclonal IgE binding of allergic patients.

**Abbreviations**
AIT: allergen‐specific immunotherapy
BSA: bovine serum albumin
PBMC: peripheral blood mononuclear cell
ScFv: single‐chain variable fragment

## 1 INTRODUCTION

Allergen‐specific immunotherapy (AIT) is an effective, disease‐modifying, and long‐lasting treatment for IgE‐associated allergy.^1^, ^2^, ^3^ AIT has been conducted originally by vaccination with natural allergen extracts containing allergens in their native structure.^4^ To increase the safety of AIT, denatured allergen extracts termed allergoids have been introduced in which the native allergens have been chemically modified to lose their structure and conformational IgE epitopes.^5^, ^6^ Despite the loss of the native structure, allergoids seem to induce allergen‐specific blocking IgG antibodies^7^, ^8^, ^9^, ^10^ which are thought to be responsible for clinical efficacy of AIT besides alterations of cellular and cytokine responses.^3^ With the availability of recombinant allergens and DNA as well as peptide‐based technologies, recombinant and synthetic allergen derivatives have been engineered for several important allergens which resemble many features of allergoids, in particular reduced IgE reactivity and reduced allergenic activity.^11^, ^12^ Several clinical studies performed with such recombinant allergen derivatives and synthetic allergen fragments lacking the folding of natural allergens indicate that the treatment is clinically effective and is associated with the induction of allergen‐specific IgG antibodies which compete with allergic patients′ IgE for binding to the natural, native allergens.^13^, ^14^, ^15^, ^16^

Although IgG antibodies induced with recombinant hypoallergenic allergen derivatives and allergen‐derived peptides are raised against unfolded allergen derivatives, they seem to induce IgG antibodies in patients which recognize also the folded native allergens. For example, immunization with recombinant, unfolded fragments of the major birch pollen allergen Bet v 1 raised IgG antibodies against Bet v 1 peptides,^17^ but serum from the immunized patients also inhibited binding of allergic patients IgE antibodies against conformational epitopes. In this context, an important question remains to be answered: Is the inhibition of allergic patients′ IgE antibodies recognizing conformational epitopes on the native allergens due to a fraction of IgG antibodies which recognizes conformational epitopes or can the inhibition of IgE antibodies be achieved with IgG antibodies which are primarily induced against peptide epitopes which also recognized the native allergen? To investigate IgG antibodies induced with recombinant unfolded allergen derivatives in detail, we immunized nonallergic subjects with recombinant Bet v 1 fragments and constructed a combinatorial single‐chain fragment library from an immunized subject who developed IgG antibodies recognizing the native allergen. We then isolated an IgG‐derived Bet v 1‐specific ScFv (H3‐1) and analyzed the sequence of H3‐1 in comparison with ancestor germline sequences, investigated the specificity, cross‐reactivity, and affinity of H3‐1. Our results demonstrated that IgG antibodies induced with recombinant, unfolded allergen derivatives simultaneously recognize peptide epitopes as well as epitopes on the folded native allergen and thus can act as blocking antibodies.

## 2 METHODS

### 2.1 Materials

Materials, sera, and reagents are described in the Data S1.

### 2.2 Construction of a combinatorial phage‐displayed ScFv library from PBMCs of a subject immunized with rBet v 1 fragments

Peripheral blood mononuclear cells (PBMCs) were obtained from a nonallergic subject who was immunized with an equimolar mixture of hypoallergenic Bet v 1 fragments^18^ adsorbed to aluminum hydroxide in the course of a single‐center, randomized, double‐blind placebo‐controlled clinical safety trial (trial number NCT01353924) (Marth and Valenta, unpublished data). This trial was conducted at the Vienna Challenge Chamber with the ethics approval of the Austrian Consortium for Clinical Pharmacology and Therapy. The subject gave written informed consent. The subjects’ IgG to Bet v 1 (2 μg/mL), Bet v 1 fragments (1 μg/mL), or peptides (1 μg/mL) was determined by ELISA. After Bet v 1, fragments or peptides were coated for 5 hours at room temperature to ELISA plates (Nunc Maxi‐Sorp, Roskilde, Denmark), plates were blocked with PBST containing 2% BSA (wt/vol) overnight at 4°C. Serum samples diluted 1:100 were incubated overnight at 4°C, and specific IgG levels were detected with a rabbit anti‐human IgG antiserum (Jackson Immuno) followed by a horseradish peroxidase‐labeled donkey anti‐rabbit antiserum (GE Healthcare, Little Chalfont, UK) diluted 1:10 000 and 1:2000, respectively, in PBST containing 0.5% BSA (wt/vol). Color reaction was read at 405 nm, and results are means of duplicates.

**Figure 1:**
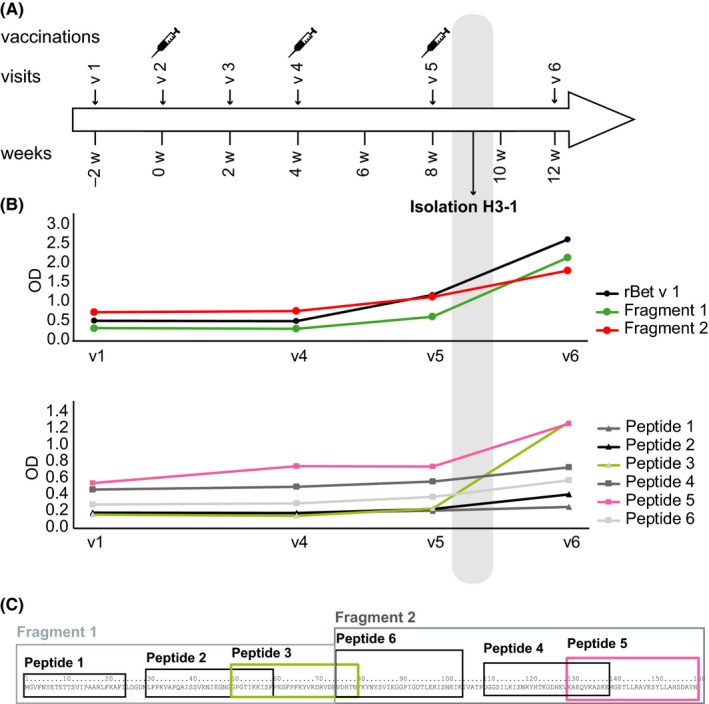
A, Scheme of immunizations. Time course (w, week) of visits (v) 1‐6 and immunizations are shown. The time point of the isolation of PBMCs for the construction of the combinatorial library leading to the isolation of ScFv H3‐1 10 days after the third immunization is indicated. B, Development of IgG responses (*x*‐axis: visits; *y*‐axis: mean OD values corresponding to IgG levels) to Bet v 1 and Bet v 1 fragments (top) or Bet v 1 peptides (bottom) in serum of the immunized subject. C, Recombinant Bet v 1 fragments and synthetic Bet v 1‐derived peptides indicated in the Bet v 1 amino acid sequence

Peripheral blood mononuclear cells were isolated 10 days after the third injection when allergen‐specific IgG had developed (Figure 1) by Ficoll Paque (GE Healthcare) density gradient centrifugation for the construction of the combinatorial library (see Data S1).

### 2.3 Isolation of a Bet v 1‐specific ScFv

After library transformation in *E. coli* TG1, phages were rescued with M13KO7 helper phage and the phage library was subjected to four rounds of solid phase panning. For this purpose, ELISA wells (Nunc, Roskilde, Denmark) were coated with Bet v 1 (1 μg/well in 1xPBS), blocked, and incubated with the phage library. Wells were washed with 1xPBS + 0.05% (vol/vol) Tween 20, and bound phages were eluted with 50 μL 100 mmol/L glycine‐HCl pH 2.2, neutralized with 3 μL 2 mol/L Tris‐HCl pH 8 and used for reinfection of a new TG1 culture that served as a starting point for the next round of panning. To check for enrichment of Bet v 1‐specific phage pools, we added each eluted phage pool from each round of panning in duplicates to ELISA plate‐bound Bet v 1 and BSA as control (10 μg/mL) and detected bound phages with horseradish peroxidase‐labeled anti‐M13 antiserum (GE Healthcare). Color reactions were read at 405 nm. Results represent means of duplicate determinations.

After the 3rd and 4th round of panning, an aliquot of transduced TG1 was plated to SOBAG plates for screening purposes (Amersham Biosciences). To this aim, single‐phage clones were rescued, incubated with ELISA plate‐bound Bet v 1 (10 μg/mL), and detected with an anti‐M13 antiserum (GE Healthcare) as described for the panning. Bet v 1‐specific phage clones were sequenced (Eurofins Genomics). Obtained sequences were aligned with human germline gene sequences from the IMGT database using V‐Quest software.^19^ Ancestor germline genes were determined, and the positions of mutations were identified. Phagemid DNAs were transformed into the nonsuppressor *E. coli* strain HB2151 for expression of soluble ScFvs carrying a peptide E tag at the C‐terminus.^20^ Additionally, ScFvs carrying a C‐terminal His tag were constructed as described.^21^ Soluble Bet v 1‐specific ScFvs were purified by affinity chromatography.^22^ The biochemical and structural characterization of H3‐1 is described in the Data S1.

### 2.4 Reactivity of H3‐1 with Bet v 1‐related allergens, rBet v 1 fragments, and Bet v 1‐derived peptides

rBet v 1, Bet v 1‐related allergens (Aln g 1, Cor a 1, Mal d 1), rBet v 1 fragments,^18^ six synthetic Bet v 1‐derived peptides^23^ or BSA (4 μg/mL in 1xPBS) were coated onto ELISA plates (Nunc), and after blocking (1xPBS/3% BSA wt/vol), 1 μg/mL H3‐1 was added. Bound ScFv was traced with an anti‐E Tag antibody (Amersham Biosciences) and detected with horseradish peroxidase‐labeled sheep anti‐mouse antiserum (GE Healthcare). All determinations were performed in duplicates with a variation of <10%, and mean values were calculated.

### 2.5 Surface representation of peptides on the three‐dimensional structure of Bet v 1

For visualization of the recognized peptide 5, a surface representation was performed. Therefore, the Bet v 1 NMR structure (PDB ID: 1B6F) was acquired from the National Center for Biotechnology Information Molecular Modeling Database, the structure was visualized with the Cn3D 4.3 software,^24^ and peptide 5 was highlighted with the sequence alignment viewer tool. For comparison, epitopes of three Bet v 1‐specific monoclonal antibodies which inhibited patients’ IgE binding to Bet v 1 have also been highlighted.^25^

### 2.6 Determination of kinetics and binding affinities by surface plasmon resonance measurements

Surface plasmon resonance measurements were performed on a Biacore 2000 (GE Healthcare) at 25°C. To activate the surface of a CM5 sensor chip flow cell (GE Healthcare), a 1:1 mixture of 1‐ethyl‐3‐(3‐dimethylaminopropyl carbodiimide) hydrochloride and N‐hydroxysuccinimide was injected at a flow rate of 5 μL/min for 7 minutes. Capturing levels of Bet v 1, Aln g 1, Cor a 1, or Mal d 1 were chosen to obtain a maximal response of 100 RU (resonance units). Allergens were diluted in 10 mmol/L sodium acetate (pH 4.5) according to pH scouting and injected into the individual flow cells at a flow rate of 5 μL/min. The reference cell was immobilized with BSA in 10 mmol/L sodium acetate (pH 5.0). The surface of the chip was deactivated by injecting 1 mol/L ethanolamine‐HCl (pH 8.5) at a flow rate of 5 μL/min for 7 minutes. Bet v 1‐specific ScFv diluted in HBS‐EP (0.01 mol/L HEPES, 0.15 mol/L NaCl, 3 mmol/L EDTA, 0.005% vol/vol surfactant P20, pH 7.4) was injected in twofold increasing concentrations into the flow cells in random order at a flow rate of 30 μL/min. Dissociation was investigated by injecting HBS‐EP for 30 minutes at 30 μL/min. Regeneration of the chip surface was established by injecting 10 mmol/L glycine‐HCl (pH 2.0) for 30 seconds at 20 μL/min. Dissociation constants and rate constants (on‐rate, off‐rate) were calculated with BIAevaluation software 3.2 (GE Healthcare) using a 1:1 interaction model.

### 2.7 Multiple sequence alignment

The amino acid sequences of Bet v 1 (Swissprot accession number: P15494), Aln g 1 (P38948), Cor a 1 (Q08407), and Mal d 1 (Q9SYW3) that correspond to the Bet v 1‐derived peptide 5 were aligned with the multiple sequence alignment tool ClustalW edited and visualized with GeneDoc.

### 2.8 Inhibition ELISA

ELISA plates (Nunc) were coated with 1 μg/mL of the allergens (Bet v 1, Aln g 1, Cor a 1, or Mal d 1), blocked, and incubated with 20 μg/mL H3‐1 or a control ScFv. After washing, sera from 30 birch pollen–allergic patients diluted 1:5 were added to the pre‐incubated plates and bound patients’ IgE antibodies were detected with alkaline phosphatase‐conjugated mouse anti‐human IgE antiserum (BD Pharmingen, San Diego, CA, USA). Sera displaying specific IgE reactivity OD<0.100 (=double value of IgE reactivity of a nonallergic) were not included in the inhibition calculation. Percentage reductions of patients’ IgE binding after pre‐incubation with H3‐1 compared to the control ScFv were calculated as follows: 100‐(OD after pre‐incubation with H3‐1 * 100/OD after pre‐incubation with control ScFv). All determinations were performed in duplicates, and median values were calculated.

Statistically significant differences of IgE binding after pre‐incubation with H3‐1 versus pre‐incubation with control ScFv were determined by Mann‐Whitney test using Prism 5.04 (GraphPad Software, La Jolla, CA, USA).

### 2.9 Effect of H3‐1 on Bet v 1‐induced basophil activation

The effect of H3‐1 on Bet v 1‐induced basophil activation was measured by detecting CD203c upregulation as described^26^ (Data S1).

## 3 RESULTS

### 3.1 Construction of a combinatorial phage‐displayed ScFv library from a subject immunized with rBet v 1 fragments and isolation of a Bet v 1‐specific ScFv

Figure 1 provides an overview of the development of IgG responses to Bet v 1, Bet v 1 fragments, and Bet v 1‐derived synthetic peptides in a subject immunized with Bet v 1 fragments. The subject had received three subcutaneous immunizations with Bet v 1 fragments in 4‐week intervals (Figure 1A). PBMCs for the construction of the combinatorial library were obtained 10 days after the third immunization. At this time point, the subject had developed increases in IgG antibody levels specific for Bet v 1 > Fragment 2 > Fragment 1 (Figure 1B). A detailed analysis with synthetic Bet v 1‐derived peptides (Figure 1C) showed that the IgG response induced by the immunization was mainly directed against the C‐terminal peptide 5 and peptide 3 which reportedly is part of a region on Bet v 1 containing binding sites for allergic patients IgE.^23^

As the IgG response was mainly composed of Bet v 1‐specific IgG_1_ and Bet v 1‐specific IgG_4_ (data not shown), preselection for IgG was achieved with specific primers located in the hinge regions of IgG_1_ or IgG_4_. VH and VΚ regions were amplified, randomly combined with DNA coding for a ((Gly_4_Ser)_3_) linker, and cloned into the phagemid vector pCANTAB 5E, leading to a final library size of 9.9 × 10^7^ independent clones. We analyzed 13 randomly picked clones to check the diversity of the library. The heavy and light chains of the analyzed clones revealed high differences in their CDR regions and in their V gene family distribution demonstrating that each clone represented an independent antibody (Data S1, Table S1), confirming the nonredundant nature of the library. The VH family distribution was dominated by members of the largest gene subgroup, the VH3 family, followed by VH1 > VH6 > VH2/VH4 > VH5 (Data S1, Table S1). The VK family distribution was dominated by VK3 followed by VK1/4 > VK7 > VK2/5/6 (Table S1).

Figure S1 shows that four rounds of bio‐panning to Bet v 1 led to a strong enrichment of Bet v 1‐positive phage especially from the second to the third and fourth round of panning. After the third round of panning, 322 phages clones were picked but none of those reacted with Bet v 1. Therefore, 401 clones were analyzed after the fourth round of panning of which 19 were found to react with Bet v 1. The 19 ScFv‐displaying phage clones were then converted into soluble ScFvs of which 15 remained reactive with Bet v 1 in their soluble form. According to sequence analysis, all these 15 clones were identical and therefore designated as one clone, that is, H3‐1.

**Figure 2:**
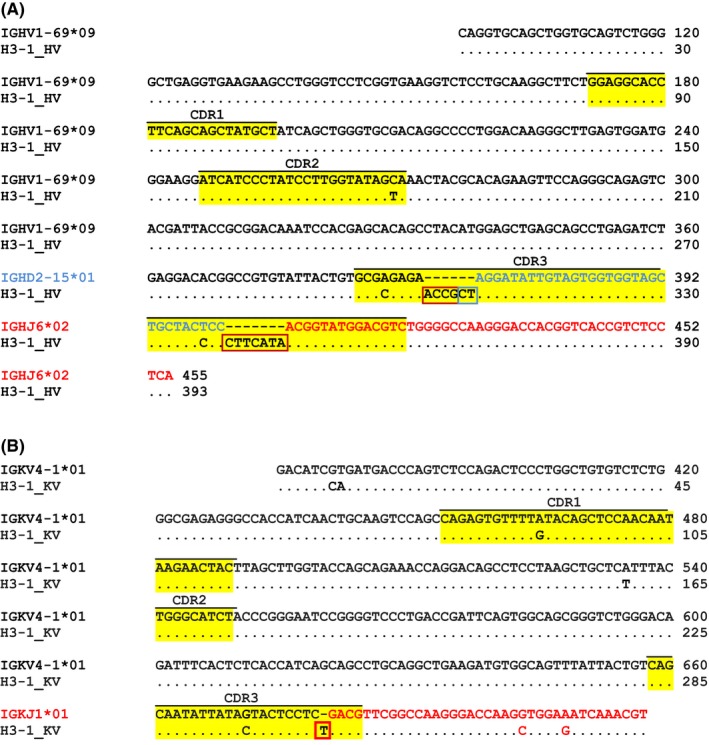
Nucleotide sequence alignment of H3‐1 (A), heavy chain (H3‐1_HV), and (B), light chain (H3‐1_KV) with the closest germline precursor genes. Variable genes are printed in black, the diversity gene in blue and joining genes in red. CDR1‐3 regions are highlighted in yellow. Identical amino acids are indicated by dots and gaps are indicated by dashes. P or N nucleotides are boxed (red: N nucleotides, blue: P nucleotides)

The H3‐1 heavy chain (H3‐1_HV) showed 99.65% nucleotide sequence identity with the closest related germline ancestor V gene IGHV1‐69*09 (Figure 2A) and differed only in one nucleotide located in CDR2 and two in CDR3 resulting in two amino acid exchanges (Data S1, Table S2). The D gene of the H3‐1_HV was derived from its germline progenitor IGHD2‐15*01. The J gene showed 100% sequence identity to the germline IGHJ6*02 (Figure 2A).

The H3‐1 light chain (H3‐1_KV) had 98.32% nucleotide sequence identity with its closest related V germline progenitor gene IGKV4‐1*01 (Figure 2B) and differed in five nucleotides (FR1: 2, CDR1: 1, FR2: 1, CDR3: 1) (Figure 2B, Table S2), leading to three amino acid changes (located in FR1, FR2, CDR3) (Table S2). The J gene of the light chain seemed to originate from IGKJ1*01 (Figure 2B).

### 3.2 Allergen specificity, cross‐reactivity, and epitope specificity of H3‐1

The H3‐1 ScFv was purified by affinity chromatography and analyzed by SDS‐PAGE. Coomassie staining revealed a single 28 kDa band without any degradation products (Figure S2A). The CD spectrum of H3‐1 showed a secondary structure dominated by ß‐sheet and ß‐turn/random coils and no trace of an α‐helical signal (Figure S2B).

**Figure 3:**
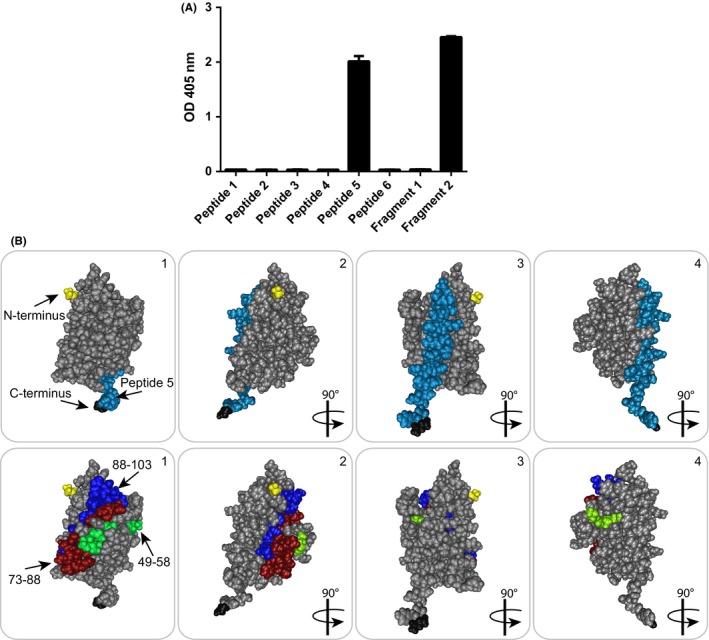
A, Reactivity (*y*‐axis: OD values ± SD) of H3‐1 to two Bet v 1 fragments and six Bet v 1‐derived peptides. B, Top, visualization of peptide 5 (highlighted in light blue) on the three‐dimensional surface representation of Bet v 1. Bottom, localization of peptides recognized by monoclonal antibodies mAb2 (aa 49‐58, green), mAb12 (aa 88‐103, dark blue), and mAb13 (aa 73‐88, red), which strongly compete with patients’ IgE binding to Bet v 1. 1‐4 show different views of the Bet v 1 surface. The N‐terminus and C‐terminus are indicated in yellow and black, respectively

To reveal the binding site of H3‐1 on Bet v 1, epitope mapping was performed with six synthetic Bet v 1‐derived peptides covering almost the complete Bet v 1 sequence and two hypoallergenic Bet v 1 fragments (F1/F2) (Figure 1C). H3‐1 bound specifically to the C‐terminal F2 (aa 75‐160) (Figure 3A). A detailed analysis with the Bet v 1 peptides allowed to map the H3‐1 binding site on the C‐terminal peptide 5 (aa 130‐160) (Figure 3A). Figure 3B shows the localization of the binding site represented by peptide 5 (light blue) on the three‐dimensional structure of Bet v 1. Peptide 5 appeared to be located on one side of the Bet v 1 molecule, whereas a major binding site mapped for allergic patients IgE antibodies as defined by three peptides recognized by blocking monoclonal antibodies (ie, aa 49‐58, aa 73‐88, and aa 88‐103) appeared to be located on the opposite side of Bet v 1 (Figure 3B).

**Figure 4:**
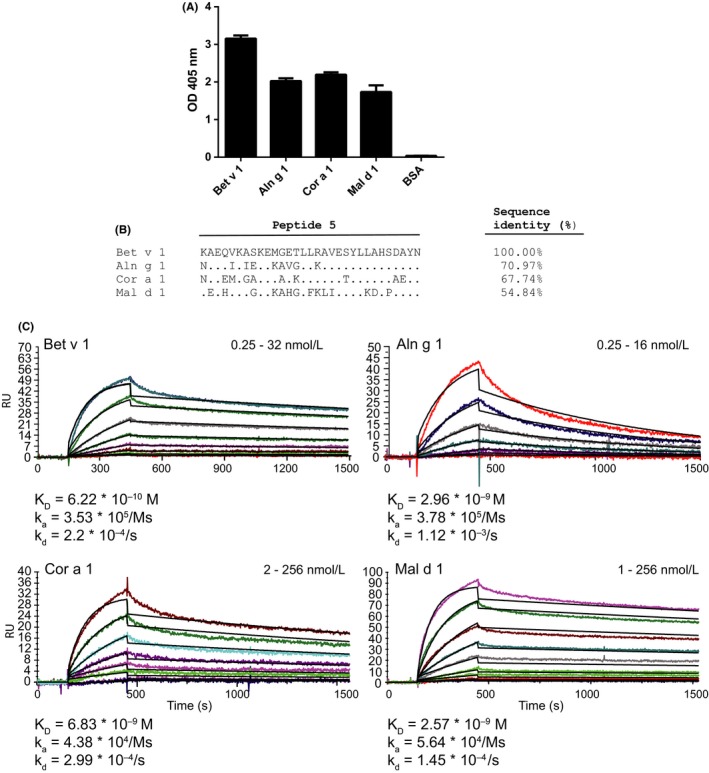
A, Reactivity (*y*‐axis: OD values ± SD) of H3‐1 to recombinant Bet v 1, Aln g 1, Cor a 1, Mal d 1 and BSA as control (*x*‐axis) is shown. B, Sequence alignment of peptide 5 from Bet v 1 with the corresponding peptides from alder, hazel, and apple. Identical amino acids are indicated by points, and sequence identities are displayed on the right margin. C, Sensor chip‐based studies of the interaction between H3‐1 and Bet v 1, Aln g 1, Cor a 1, Mal d 1. Allergens were immobilized on the chip and H3‐1 was injected in twofold increasing concentrations indicated in the right corners of the figures (nmol/L). Resonance units (RU) are displayed (*y*‐axes) for the time course (seconds; *x*‐axes). Recorded (colored lines) and calculated (black lines) curves, which represent fitting to a 1:1 binding model, were superimposed. Dissociation constants (K_D_) as well as association and dissociation rate constants (k_a_, k_d_) are displayed

Next, we studied the cross‐reactivity of H3‐1 with Bet v 1‐related allergens. Figure 4A shows that H3‐1 reacted best with rBet v 1 but also with the cross‐reactive allergens from alder (Aln g 1), hazel (Cor a 1), and apple (Mal d 1), albeit with lower intensity. No reactivity to the control protein BSA was observed (Figure 4A). Figure 4B shows an alignment of the Bet v 1‐derived peptide 5 with the corresponding regions in Aln g 1, Cor a 1, and Mal d 1. The corresponding peptides showed sequence identities of 71% (Aln g 1), 67.7% (Cor a 1), and 54.8% (Mal d 1).

### 3.3 High‐affinity binding of H3‐1 to Bet v 1 and homologous allergens

Surface plasmon resonance measurements were conducted for H3‐1 binding to Bet v 1 and the homologous proteins Aln g 1, Cor a 1, and Mal d 1. H3‐1 bound to all four allergens with high affinities (Bet v 1: K_D_: 6.22 × 10^−10 ^M, k_a_: 3.53 × 10^5^/Ms, k_d_: 2.2 × 10^−4^/s; >Mal d 1: K_D_: 2.57 × 10^−9 ^M, k_a_: 5.64 × 10^4^/Ms, k_d_: 1.45 × 10^−4^/s; >Aln g 1: K_D_: 2.96 × 10^−9^ M, k_a_: 3.78 × 10^5^/Ms, k_d_: 1.12 × 10^−3^/s; >Cor a 1: K_D_: 6.83 × 10^−9^ M, k_a_: 4.38 × 10^4^/Ms, k_d_: 2.99 × 10^−4^/s) (Figure 4C).

### 3.4 Effects of H3‐1 on patients’ allergen‐specific IgE binding and basophil activation

Next, we tested whether H3‐1 is able to inhibit birch pollen–allergic patients’ IgE binding to ELISA plate‐bound Bet v 1 or to cross‐reactive Aln g 1, Cor a 1, and Mal d 1 in a representative number of patients (n = 30). Compared to a control ScFv with other specificity, H3‐1 caused a mean inhibition of patients’ IgE binding to Bet v 1 of 45% (0%‐62.9%) (Table 1). Patients’ IgE binding to cross‐reactive allergens was inhibited to a lower degree (ie, Cor a 1: 24.3%, 0%‐57%; Mal d 1: 13.5%, 0%‐40.5%; Aln g 1: 11.9%, 0%‐37.1%) (Table 1).

**Table 1:** Inhibition of allergic patients′ IgE binding to Bet v 1, Aln g 1, Cor a 1, and Mal d 1 after pre‐incubation with H3‐1 compared to a control ScFv

	H3‐1	Control ScFv	% Inhibition to Bet v 1	H3‐1	Control ScFv	% Inhibition to Aln g 1	H3‐1	Control ScFv	% Inhibition to Cor a 1	H3‐1	Control ScFv	% Inhibition to Mal d 1
Patient 1	0.302	0.771	60.9	0.097	0.106	8.2	0.180	0.268	32.7	0.104	0.114	8.6
Patient 2	0.243	0.563	56.8	0.067	0.083	0.0	0.049	0.048	0.0	0.125	0.163	22.8
Patient 3	1.803	2.125	15.2	1.305	1.119	0.0	0.570	0.501	0.0	0.613	0.546	0.0
Patient 4	0.305	0.583	47.7	0.167	0.195	14.0	0.082	0.087	0.0	0.069	0.070	0.0
Patient 5	1.232	1.700	27.6	1.747	1.625	0.0	0.329	0.463	29.0	2.027	2.103	3.6
Patient 6	0.161	0.424	62.1	0.066	0.086	0.0	0.067	0.096	0.0	0.181	0.259	30.2
Patient 7	0.333	0.576	42.2	0.242	0.258	6.3	0.081	0.100	19.0	0.248	0.245	0.0
Patient 8	0.264	0.569	53.7	0.190	0.256	26.0	0.066	0.078	0.0	0.064	0.065	0.0
Patient 9	0.881	1.868	52.8	0.289	0.341	15.3	0.171	0.334	48.9	0.162	0.182	10.9
Patient 10	0.696	1.193	41.7	0.816	0.807	0.0	0.677	0.741	8.6	0.434	0.485	10.5
Patient 11	0.581	1.193	51.3	0.420	0.541	22.5	0.145	0.144	0.0	0.566	0.700	19.1
Patient 12	0.502	1.302	61.4	0.300	0.351	14.5	0.138	0.250	44.9	0.422	0.545	22.4
Patient 13	0.303	0.612	50.5	0.261	0.295	11.4	0.383	0.589	35.0	0.099	0.136	27.4
Patient 14	0.102	0.141	27.5	0.064	0.064	0.0	0.085	0.095	0.0	0.046	0.044	0.0
Patient 15	0.532	0.434	0.0	0.379	0.264	0.0	0.150	0.098	0.0	0.040	0.041	0.0
Patient 16	0.781	1.537	49.2	0.254	0.304	16.3	0.120	0.148	18.4	0.161	0.165	2.3
Patient 17	1.090	2.223	51.0	0.345	0.459	25.0	0.566	0.993	43.0	0.136	0.228	40.5
Patient 18	0.502	1.180	57.4	2.502	2.342	0.0	0.456	0.773	40.9	0.113	0.137	17.1
Patient 19	1.124	2.473	54.6	0.683	0.818	16.5	0.532	0.800	33.6	0.214	0.252	15.3
Patient 20	0.379	0.451	15.8	0.382	0.411	6.9	0.413	0.625	33.9	0.085	0.102	16.5
Patient 21	0.287	0.522	44.9	0.104	0.119	13.4	0.128	0.136	6.0	0.096	0.109	11.9
Patient 22	0.767	2.065	62.9	0.132	0.210	37.1	0.487	1.052	53.7	0.087	0.119	26.3
Patient 23	1.331	2.525	47.3	1.069	1.141	6.2	0.190	0.240	21.1	0.125	0.129	3.0
Patient 24	1.152	2.067	44.3	0.846	0.971	12.9	0.197	0.308	36.0	0.258	0.320	19.3
Patient 25	0.714	1.922	62.9	0.462	0.560	17.5	0.293	0.691	57.6	0.209	0.254	17.9
Patient 26	0.554	1.121	50.6	0.371	0.486	23.7	0.087	0.111	22.0	0.286	0.356	19.7
Patient 27	0.563	1.486	62.1	1.744	2.359	26.1	0.124	0.181	31.2	0.093	0.127	27.4
Patient 28	1.786	0.882	0.0	0.643	0.870	26.1	1.928	3.513	45.1	0.650	0.708	8.2
Patient 29	0.197	0.323	38.8	0.068	0.071	0.0	0.181	0.300	39.6	0.095	0.123	22.5
Patient 30	0.309	0.715	56.8	0.414	0.464	10.7	0.188	0.266	29.5	0.073	0.070	0.0
Nonallergic	0.058	0.060		0.048	0.049		0.084	0.088		0.049	0.050	
Mean			45.0			13.2			29.1			16.1

ELISA plate‐bound allergens had been pre‐incubated with H3‐1 or a control ScFv and subsequently exposed to patients sera. The OD values corresponding to IgE reactivity to the allergens are displayed and are means of duplicates. Percentage reduction of patients’ IgE binding after pre‐incubation with H3‐1 compared to the control ScFv to Bet v 1, Aln g 1, Cor a 1, and Mal d 1, is displayed in the 4th, 7th, 10th and 12th column, respectively.

**Figure 5:**
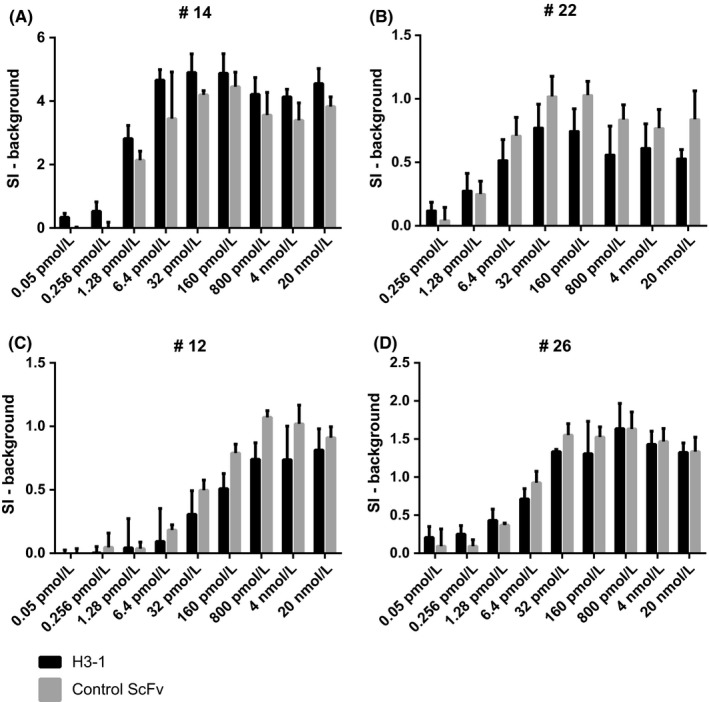
Effect of H3‐1 on Bet v 1‐induced basophil activation. Blood samples from four birch pollen–allergic patients (A: #14; B: #22; C: #12, D: #26) were exposed to increasing doses of Bet v 1 (*x*‐axes) which had been pre‐incubated with H3‐1 (black bars) or a control ScFv (gray bars). Upregulation of CD203c expression on basophils was determined by FACS analyses and is displayed as stimulation index (SI) minus background (ie, SI observed with ScFvs only) for each patient (*y*‐axes: SI) ±SD

A varying effect of H3‐1 on Bet v 1‐induced basophil activation was observed (Figure 5A‐D). In three of the four patients examined, H3‐1 inhibited Bet v 1‐induced in vitro basophil activation. An approximately fivefold reduction was observed for patients #12 and #26 (Figure 5D,C) and approximately 25‐fold reduction for patient #22 (Figure 5B). For patient #14 (Figure 5A), it appeared that H3‐1 augmented basophil activation approximately fivefold.

The effects of H3‐1 on IgE binding of these patients were as follows: inhibition of IgE binding #14: 27.5%; #22: 62.9%; #26: 50.6%; #12: 61.4% (Table 1).

## 4 DISCUSSION

Our study is the first to report the molecular characterization of an allergen‐specific IgG antibody induced by immunization with recombinant hypoallergenic allergen derivatives in a nonallergic subject. An IgG‐derived Bet v 1‐specific ScFv (H3‐1) was isolated from a combinatorial library constructed from a subject immunized with recombinant hypoallergenic fragments of the major birch pollen allergen Bet v 1. Although the recombinant Bet v 1 fragments are unfolded proteins, we found that immunization induced a strong IgG response not only against each of the fragments but also against the folded, native allergen (Figure 1). H3‐1 was isolated from PBMCs at a time point after immunization when, according to earlier work, affinity maturation had occurred and thus should have given rise to antibodies showing extensive somatic mutations in the variable regions of the heavy and light chains.^27^ However, sequence analysis of H3‐1 revealed that it contained only few mutations in the variable regions and thus appeared to be almost in germline configuration. So far, only few allergen‐specific IgG antibodies induced by AIT have been described. The earlier described Bet v 1‐specific IgG antibodies BAB1‐5 and a grass pollen allergen (ie, Phl p 7)‐specific IgG antibody mAb102.1F10 were isolated from allergic patients who had received AIT with natural, native allergen‐containing extracts.^28^, ^29^ In contrast to H3‐1, these antibodies showed numerous somatic mutations and signs of affinity maturation.

Nevertheless, we found that H3‐1 bound with high affinity to native and folded Bet v 1 as well as to Bet v 1‐related allergens. This finding is remarkable because high‐affinity binding was so far considered a feature of highly mutated antibodies.^30^ Only a few antibodies binding with high affinity without showing extensive signs of somatic hypermutation have been described such as an IL‐17 neutralizing antibody,^31^ antiviral IgG antibodies specific for VSV,^32^ and anti‐influenza haemagglutinin IgG antibodies.^33^ Interestingly, H3‐1 recognized not only folded Bet v 1 but also a synthetic, unfolded peptide derived from the C‐terminus of Bet v 1. H3‐1 is thus an example to show that IgG antibodies that were induced in allergic subjects who had been immunized with recombinant Bet v 1 fragments also bind to folded native Bet v 1 allergen.^13^, ^17^ The polyclonal IgG response induced in these patients with Bet v 1 fragments was also directed against synthetic Bet v 1‐derived peptides, among them the peptide recognized by H3‐1 and against the folded native Bet v 1 allergen. Thus, it was not possible to decipher in the earlier clinical study what fraction of the patients’ IgG response actually contributed to the inhibition of polyclonal IgE binding to conformational Bet v 1 epitopes. Using H3‐1, we were now able to demonstrate that a single monoclonal IgG antibody induced by immunization against a peptide epitope of Bet v 1 not only recognizes native, folded Bet v 1 but also inhibits polyclonal allergic patients' IgE specific for conformational epitopes on Bet v 1. The fact that only one single ScFv was obtained from the immunized subject is certainly a limitation of our study. Nevertheless, it may be of clinical relevance because it exemplifies that vaccination and AIT with recombinant and/or synthetic unfolded allergen derivatives are able to induce blocking IgG antibodies which also recognize the native allergen.^13^, ^14^, ^15^ It thus provides an explanation for the efficacy of recombinant and synthetic allergen derivatives in AIT. Our observation may also explain why denatured allergen extracts (ie, allergoids) which are currently used in clinical practice for AIT can induce IgG antibodies against the wild‐type allergens and thus provide a mechanism for the mode of activity of allergoids in AIT.^7^, ^8^, ^9^, ^10^ Finally, promising results have been obtained in AIT studies with a new type of allergy vaccines which are based on carrier‐bound allergen‐derived peptides which induce mainly peptide‐specific IgG antibodies which also strongly inhibit polyclonal IgE binding of allergic patients toward conformational epitopes.^16^, ^34^, ^35^ The effects of these B cell epitope‐based allergy vaccines may also be explained by induction of peptide‐specific IgG antibodies which can block allergic patients' IgE binding to the natural allergen.

## AUTHORS CONTRIBUTIONS

Elisabeth Gadermaier involved in the concept and design, acquisition of data, analysis, and interpretation of data, drafted the article, and finally approved the version to be published. Katharina Marth, Christian Lupinek, Raffaela Campana, Gerhard Hofer, Katharina Blatt, Dubravka Smilijkovic, Uwe Roder, Margarete Focke‐Tejkl, Susanne Vrtala, Walter Keller, and Peter Valent involved in acquisition of data, analysis and interpretation of data, critically revised the article, and finally approved the version to be published. Rudolf Valenta involved in study concept and design, analysis, and interpretation of data, critically revised the article, and gave final approval of the version to be published. Sabine Flicker involved in study concept and design, analysis, and interpretation of data, drafted and critically revised the article, and gave final approval of the version to be published.

## CONFLICTS OF INTEREST

Rudolf Valenta has received research grants from BIOMAY AG, Vienna, Austria, and Viravaxx, Vienna and serves as a consultant for these companies.
