# Supplementary material for: Isolation of a high‐affinity Bet v 1‐specific IgG‐derived ScFv from a subject vaccinated with hypoallergenic Bet v 1 fragments
Source: Allergy. 2018 Feb 20;73(7):1425–35. doi: 10.1111/all.13394 (PMC6032869; doi:10.1111/all.13394)
Supplement: Supplementary file 3 [file ALL-73-1425-s003.docx]

|  | Heavy chain | | | |  | Light chain |
| --- | --- | --- | --- | --- | --- | --- |
|  | CDR1 | CDR2 | CDR3 | IGHV |  | IGKV |
| **1** | GFSHSDARMG | IFSNDEK-- | ARME-EQQRD----AFDI--- | IGHV2-26*01 |  | IGKV4-1*01 |
| **2** | ..TF.N--YW | VN-S.GTDA | ..VAYSNSGA----F..Y--- | IGHV3-74*01 or *02 or *03 |  | IGKV1-39*01 or IGKV1D-39*01 |
| **3** | .HTFTS--H. | SS-GYNVNP | .SHRSYYNFWSGPGW..P--- | IGHV1-18*01 |  | n.a. |
| **4** | .YTFTG--NY | VN-P.SVGT | ..DLGPMYSMIIVGG..S--- | IGHV1-2*02 or *04 |  | IGKV1-5*03 |
| **5** | .YIFTS--YD | ..-PG.G.T | ..GDYYR------RY..L--- | IGHV1-2*02 or IGHV1-8*01 |  | IGKV7-3*01 |
| **6** | ...FTN--YW | VN-T.GSTT | T.SGGSE--------..Y--- | IGHV3-74*02 |  | IGKV3-20*01 |
| **7** | .D.I.SNDYY | VYYTGST-- | V.QR----IA----.Y.S--- | IGHV4-39*01 |  | IGKV3-11*01 |
| **8** | .LTVK.--RY | .S-SSGFTI | .SGYSSRVN------..S--- | IGHV3-11*01 |  | IGKV1D-8*01 |
| **9** | ..TFIN--YA | LT-SAG-AT | ..ELGRTG-------.TPLTT | IGHV3-23*01 or *04 or D*01 |  | IGKV4-1*01 |
| **10** | RD.L.T--YT | .I-PLFGTA | ..VTSSHSPR-FLYYM..--- | IGHV1-69*01 or D*01 |  | n.a. |
| **12** | SP.VVT---. | ---HVSVVM | IIV-GGRRVLLC-----Y--- | IGHV3-74*01 or *03 |  | IGKV4-1*01 |
| **14** | .D.V.SNNAA | TYFRSKWYH | G.SDYANSKW----Y..L--- | IGHV6-1*01 |  | IGKV3-NL5*01 or IGKV3D-20*01 |
| **15** | .D.V.AHSAI | TYYTSRWY. | T.------------WVGD--- | IGHV6-1*01 |  | IGKV3-20*01 |

Table S1

Diversity check of randomly picked clones

n.a. not available
