# Supplementary material for: Isolation of a high‐affinity Bet v 1‐specific IgG‐derived ScFv from a subject vaccinated with hypoallergenic Bet v 1 fragments
Source: Allergy. 2018 Feb 20;73(7):1425–35. doi: 10.1111/all.13394 (PMC6032869; doi:10.1111/all.13394)
Supplement: Supplementary file 4 [file ALL-73-1425-s004.docx]

**Table S2**

A. Mutation analysis of the variable heavy chain region from H3-1

|  | | **IGHV** | **FR1** | **CDR1** | **FR2** | **CDR2** | **FR3** | **CDR3** |
| --- | --- | --- | --- | --- | --- | --- | --- | --- |
| **Nucleotides** | | 354 | 75 | 24 | 51 | 24 | 114 | 66 |
| **Identical nucleotides** | | 351 | 75 | 24 | 51 | 23 | 114 | 64 |
| **Mutations** | | 3 | 0 | 0 | 0 | 1 | 0 | 2 |
| **Mutations** | **Silent** | 1 | 0 | 0 | 0 | 0 | 0 | 1 |
|  | **Nonsilent** | 2 | 0 | 0 | 0 | 1 | 0 | 1 |

|  | **IGHV** | **FR1** | **CDR1** | **FR2** | **CDR2** | **FR3** | **CDR3** |
| --- | --- | --- | --- | --- | --- | --- | --- |
| **AA** | 118 | 25 | 8 | 17 | 8 | 38 | 33 |
| **Identical AA** | 116 | 25 | 8 | 17 | 7 | 38 | 32 |
| **AA changes** | 2 | 0 | 0 | 0 | 1 | 0 | 1 |

B. Mutation analysis of the variable light chain region from H3-1

|  | | **IGKV** | **FR1** | **CDR1** | **FR2** | **CDR2** | **FR3** | **CDR3** |
| --- | --- | --- | --- | --- | --- | --- | --- | --- |
| **Nucleotides** | | 309 | 78 | 36 | 51 | 9 | 108 | 27 |
| **Identical nucleotides** | | 303 | 76 | 35 | 50 | 9 | 108 | 25 |
| **Mutations** | | 6 | 2 | 1 | 1 | 0 | 0 | 2 |
| **Mutations** | **Silent** | 2 | 0 | 1 | 0 | 0 | 0 | 1 |
|  | **Nonsilent** | 4 | 2 | 0 | 1 | 0 | 0 | 1 |

|  | **IGKV** | **FR1** | **CDR1** | **FR2** | **CDR2** | **FR3** | **CDR3** |
| --- | --- | --- | --- | --- | --- | --- | --- |
| **AA** | 103 | 26 | 12 | 17 | 3 | 36 | 9 |
| **Identical AA** | 100 | 25 | 12 | 16 | 3 | 36 | 6 |
| **AA changes** | 3 | 1 | 0 | 1 | 0 | 0 | 1 |
