# Supplementary material for: Isolation of a high‐affinity Bet v 1‐specific IgG‐derived ScFv from a subject vaccinated with hypoallergenic Bet v 1 fragments
Source: Allergy. 2018 Feb 20;73(7):1425–35. doi: 10.1111/all.13394 (PMC6032869; doi:10.1111/all.13394)
Supplement: Supplementary file 5 [file ALL-73-1425-s005.docx]

**Data S1**

**Isolation of a high affinity Bet v 1-specific IgG-derived ScFv from a subject vaccinated with hypoallergenic Bet v 1 fragments**

Elisabeth Gadermaier^1^, Katharina Marth^1^, Christian Lupinek^1^, Raffaela Campana^1^, Gerhard Hofer^2^,Katharina Blatt^3^, Dubravka Smiljkovic^3^, Uwe Roder^4^, Margarete Focke-Tejkl^1^, Susanne Vrtala ^1^, Walter Keller^2^, Peter Valent^3^, Rudolf Valenta^1^, Sabine Flicker^1^.

^1^Division of Immunopathology, Institute of Pathophysiology and Allergy Research, Center for Pathophysiology, Infectiology and Immunology, Medical University of Vienna, Vienna General Hospital, Vienna, Austria.

^2^Institute of Molecular Biosciences, University of Graz, Graz, Austria

^3^Division of Hematology and Hemostaseology, Department of Internal Medicine I, Medical University of Vienna, Vienna General Hospital, Vienna, Austria.

^4^GE Healthcare Europe GmbH, Freiburg, Germany.

Correspondence to:

Sabine Flicker

Division of Immunopathology

Department of Pathophysiology and Allergy Research

Center for Pathophysiology, Infectiology and Immunology

Medical University of Vienna, Vienna General Hospital

Währinger Gürtel 18-20, A-1090 Vienna, Austria

Tel.: +43-1-40400-51150

Fax: +43-1-40400-51300

e-mail: sabine.flicker@meduniwien.ac.at

METHODS

**Recombinant allergens, recombinant allergen fragments, synthetic peptides, extracts, patients’ sera**

Recombinant allergens, Bet v 1 (birch), Aln g 1 (alder), Cor a 1 (hazel) and Mal d 1 (apple) were purchased from Biomay (Vienna, Austria). Synthetic peptides spanning the whole Bet v 1 sequence were synthesized on an Applied Biosystems peptide synthesizer, Model 433A (Foster City, California, USA) and purified as described^E1^. Recombinant Bet v 1 fragments covering amino acids 1 – 74 (fragment 1) and amino acids 75 – 160 (fragment 2) were expressed in *Escherichia coli* and purified as described^E2^.

Sera and heparinized blood samples were obtained from patients suffering from birch pollen allergy according to case history, skin prick testing and IgE serology after informed consent was obtained. Serum and blood samples were analyzed in an anonymized manner with permission from the Ethics committee of the Medical University of Vienna (EK641/2014).

**Construction of a combinatorial phage-displayed ScFv library**

RNA was prepared from the PBMCs by guanidinium isothiocyanate treatment and cesium chloride density gradient centrifugation. Using a mix of IgG_1_- and IgG_4_-specific primers located in the antibody hinge region (IgG_1_-primer: 5’ GCA TGT ACT AGT TTT GTC ACA AGA TTT GGG 3’; IgG_4_-primer: 5’ GCA TGA ACT AGT TGG GGG ACC ATA TTT GGA 3’) RNA was transcribed into gamma heavy chain variable region cDNA (SuperScript TM First-Strand Synthesis System for RT-PCR, Invitrogen, Carlsbad, CA, USA) according to the manufacturer’s protocol. In PCR reactions with IGHV and a pool of IGHJ family-specific primers^E1^ sequences coding for individual V gene families of heavy chain variable regions were obtained (GoTaq Green Master Mix, Promega, WS, USA). In order to isolate light chains, cDNA was first generated with oligo(dT) primers and in a subsequent PCR step with IGKV and a pool of IGKJ family-specific primers ^E3^ sequences coding for individual families of kappa light chain variable regions were amplified. IGHV and IGKV sequences from all families were separated on 1% agarose gel, eluted (Wizard SV gel and PCR Clean Up System, Promega, WS, USA) and pooled. In an assembly PCR reaction (Amersham Biosciences, Little Chalfont, United Kingdom), pools of heavy and light chain family variable sequences together with linker DNA coding for a 15 amino acids linker ((Gly_4_Ser)_3_) were merged and further amplified with IGHV- and IGKJ-specific primers containing restriction sites for the endonucleases *SfiI* and *NotI*.^E4^ Assembled products were again separated on a 1% agarose gel and purified by gel elution. Products were digested with *Sfi*I and *Not*I (New England Biolabs, Ipswich, Mass, USA), purified with a reaction clean up kit (MinElute Reaction Cleanup Kit, Qiagen, Germany) and ligated into the *Sfi*I and *Not*I-digested phagemid vector pCANTAB 5 E (Amersham Biosciences, Little Chalfont, United Kingdom). Competent *E. coli* cells of the suppressor strain TG1, which produce ScFv-g3p fusion proteins that can be displayed on the M13 phage after rescue, were produced and transformed with the ScFv pool ligated in pCANTAB 5 E by electroporation. To determine the library size and to check the diversity of the library, transformed bacteria were plated on SOBAG-plates (Amersham Biosciences) containing ampicillin and incubated at 30°C overnight. Thirty colonies were picked, the corresponding phagemid DNAs were prepared (Wizard Plus SV Minipreps DNA Purification System, Promega), sequenced (Eurofins Genomics, Ebersberg, Germany) and analyzed to exclude bias in the library. Therefore, DNA sequences were translated (ExPASy, Swiss Institute of Bioinformatics, Geneva, Switzerland) and amino acid sequences were aligned with the multiple sequence alignment tool ClustalW (European Bioinformatics Institute, Cambridge, UK). Further, ancestor germline genes were determined with the IMGT database using V-Quest software.^E5^

**SDS-PAGE and circular dichroism analysis of purified ScFv H3-1**

The purity of H3-1 was determined by SDS-PAGE under reducing conditions followed by staining with Coomassie brilliant blue. Circular dichroism (CD) measurements were performed on a Jasco J-1500 spectropolarimeter (Jasco, Gross-Umstadt, Germany)^E6^. Spectra were recorded from 190 nm to 260 nm at 20 nm/min in a 0.1 mm cell with 2 nm bandwidth and 4 seconds integration time. H3-1 was measured at a concentration of 0.4 mg/ml at room temperature. Ten individual spectra were averaged and the average of 10 identically collected buffer (40mM NaCl, 20 mM Tris pH 8.2) scans were subtracted. The results were expressed as the mean residue ellipticity.

**Effect of H3-1 on Bet v 1-induced basophil activation**

The effect of H3-1 on Bet v 1-induced basophil activation was measured by detecting CD203c up-regulation as described^E7^. Heparinized blood samples (100µl) were incubated for 15 minutes at 37°C with increasing concentrations of allergens (Bet v 1, Aln g 1, Cor a 1, Mal d 1; 0.256 pM – 20 nM) that had been pre-incubated overnight at 4°C with 20 µg/ml of either H3-1 or control ScFv. For control purposes, cells were exposed to 1) the ScFvs (20µg/ml) without allergen addition, 2) to 1µg/ml of a monoclonal anti-IgE antibody (Immunotech, Marseille, France) or 3) to buffer alone. CD203c expression levels were determined by flow cytometry^E7^. Allergen-induced CD203c up-regulation was calculated from mean fluorescence intensities (MFIs) obtained with stimulated (MFI_stim_) and unstimulated (MFI_control_) cells and was expressed as stimulation index (SI) which is defined as MFI_stim_/MFI_control._ Mean SIs of triplicate determinations were calculated, background reactions of the ScFvs without allergen addition were subtracted and results are displayed as SIs minus background^E7^. Fold-reduction was calculated by comparing allergen quantities needed to elicit a comparable mean SI after pre-incubation with H3-1 with those needed to obtain a comparable SI after pre-incubation with the control ScFv. For example, after pre-incubation with H3-1 160pM Bet v 1 are needed to elicit a SI of 0.75 while only 6.4pM Bet v 1 are needed when pre-incubation was done with the control ScFv to reach a comparable SI (patient 22). This difference is calculated as a 25-fold reduction (160: 6.4 = 25).

E1. Focke M, Linhart B, Hartl A, et al. Non-anaphylactic surface-exposed peptides of the major birch pollen allergen, Bet v 1, for preventive vaccination. *Clin Exp Allergy* 2004;34:1525–1533.

E2. Vrtala S, Hirtenlehner K, Vangelista L, et al. Conversion of the major birch pollen allergen, Bet v 1, into two nonanaphylactic T cell epitope-containing fragments: candidates for a novel form of specific immunotherapy. *J Clin Invest*. 1997;99:1673–1681.

E3. Marks JD, Hoogenboom HR, Bonnert TP, et al. By-passing immunization. Human antibodies from V-gene libraries displayed on phage. J Mol Biol 1991;222:581–597.

E4. Madritsch C, Gadermaier E, Roder UW, et al. High-density IgE recognition of the major grass pollen allergen Phl p 1 revealed with single-chain IgE antibody fragments obtained by combinatorial cloning. J Immunol 2015;194:2069–2078.

E5. Brochet X, Lefranc M-P, Giudicelli V. IMGT/V-QUEST: the highly customized and integrated system for IG and TR standardized V-J and V-D-J sequence analysis. Nucleic Acids Res 2008;36:W503-508.

E6. Lupinek C, Roux KH, Laffer S, et al. Trimolecular complex formation of IgE, Fc epsilon RI, and a recombinant nonanaphylactic single-chain antibody fragment with high affinity for IgE. J Immunol 2009;182:4817–4829.

E7. Hauswirth AW, Natter S, Ghannadan M, et al. Recombinant allergens promote expression of CD203c on basophils in sensitized individuals. J Allergy Clin Immunol 2002;110:102–109.

FIGURES and TABLES

**FIGURE S1.** Enrichment of Bet v 1-reactive phage during the panning of the combinatorial library to Bet v 1. Binding of phage (OD values: y-axis) to Bet v 1 (circles) or to BSA (squares) (x-axis) before and after 1, 2, 3 or 4 rounds of panning to Bet v 1.

**FIGURE S2.** (A), Coomassie brilliant blue-stained SDS-PAGE of purified H3-1. Molecular masses (kDa) are shown on the left margin. (B), Circular dichroism analysis of H3-1. The mean residue ellipticity (y-axis) is displayed at different wavelengths (x-axis).

**Supplemental Table S1.** Diversity of 13 randomly picked clones of the combinatorial library. Shown are the sequences of the heavy chain CDRs using the first sequence as reference (dots represent identical amino acids, dashes indicate gaps) and the distribution of the VH and VK families.

**Supplemental Table S2.** Mutation analysis of the variable regions of the heavy (A) and light chain (B) of H3-1 is shown (junction is not included in the analysis). Sequences were analyzed at the nucleotide level (top) and amino acid (AA) level (bottom). Changes of nucleotides and amino acids as compared to germline are highlighted in grey for the different regions.
